# Amorphous Form of Carvedilol Phosphate—The Case of Divergent Properties

**DOI:** 10.3390/molecules26175318

**Published:** 2021-09-01

**Authors:** Szymon Sip, Natalia Rosiak, Andrzej Miklaszewski, Patrycja Talarska, Ewa Dudziec, Judyta Cielecka-Piontek

**Affiliations:** 1 Department of Pharmacognosy, Poznan University of Medical Sciences, 4 Swiecickiego Street, 60-781 Poznan, Poland; szymonsip@ump.edu.pl (S.S.); nrosiak@ump.edu.pl (N.R.); 2 Institute of Materials Science and Engineering, Poznan University of Technology, Jana Pawła II 24, 61-138 Poznan, Poland; andrzej.miklaszewski@put.poznan.pl; 3 Department of Immunobiology, Poznan University of Medical Sciences, ul. Rokietnicka 8, 60-806 Poznan, Poland; patrycjatalarska@ump.edu.pl; 4 Department of Rheumatology and Rehabilitation, Poznan University of Medical Sciences, ul. 28 Czerwca 1956 r. 135/147, 61-545 Poznan, Poland; ewa.dudziec@ump.edu.pl

**Keywords:** carvedilol phosphate, amorphous, solid dispersion, dissolution rate, permeability

## Abstract

The amorphous form of carvedilol phosphate (CVD) was obtained as a result of grinding. The identity of the obtained amorphous form was confirmed by powder X-ray diffraction (PXRD), different scanning calorimetry (DSC), and FT-IR spectroscopy. The process was optimized in order to obtain the appropriate efficiency and time. The crystalline form of CVD was used as the reference standard. Solid dispersions of crystalline and amorphous CVD forms with hydrophilic polymers (hydroxypropyl-β-cyclodextrin, Pluronic^®^ F-127, and Soluplus^®^) were obtained. Their solubility at pH 1.2 and 6.8 was carried out, as well as their permeation through a model system of biological membranes suitable for the gastrointestinal tract (PAMPA-GIT) was established. The influence of selected polymers on CVD properties was defined for the amorphous form regarding the crystalline form of CVD. As a result of grinding (four milling cycles lasting 15 min with 5 min breaks), amorphous CVD was obtained. Its presence was confirmed by the “halo effect” on the diffraction patterns, the disappearance of the peak at 160.5 °C in the thermograms, and the changes in position/disappearance of many characteristic bands on the FT-IR spectra. As a result of changes in the CVD structure, its lower solubility at pH 1.2 and pH 6.8 was noted. While the amorphous dispersions of CVD, especially with Pluronic^®^ F-127, achieved better solubility than combinations of crystalline forms with excipients. Using the PAMPA-GIT model, amorphous CVD was assessed as high permeable (*Papp* > 1 × 10^−6^ cm/s), similarly with its amorphous dispersions with excipients (hydroxypropyl-β-cyclodextrin, Pluronic^®^ F-127, and Soluplus^®^), although in their cases, the values of apparent constants permeability were decreased.

## 1. Introduction

Carvedilol phosphate (CVD) is a phosphate salt derivative of carbazole and propranolol, a non-cardioselective beta-blocker. CVD is used in treatment as a racemic mixture; the S (−) enantiomer is a beta-adrenoceptor, and the R (+) enantiomer is both a beta and alpha-1 adrenoceptor blocker [1]. The result of blocking β receptors reduces the stroke and cardiac output capacity, reduces oxygen consumption by the heart muscle, reduces plasma renin activity, and inhibits norepinephrine release. Inhibition of alpha-1 adrenergic receptors determines the relaxation of smooth muscles in the circulatory system and lowers blood pressure. The use of high doses of CVD causes blockage of calcium channels and determines antioxidant activity, blocking the oxidation process of low-density lipoproteins, limiting their access to the coronary circulation. Carvedilol is mainly administered in chronic symptomatic heart failure, arterial hypertension, secondary prevention of heart attacks, stable angina, and ischemic heart disease [2]. The dosage is individually adjusted for the patient; however, it starts with the lowest possible dose and gradually increases to the therapeutic dose [3,4]. An essential aspect of modifying the therapy is discontinuing beta-blockers gradually before implementing a new treatment [5].

CVD belongs to the BCS 2 group of API and shows low solubility and high permeability through cell membranes [6]. The critical aspect of improving bioavailability for this group of compounds is increasing solubility due to the lack of API absorption limit [7,8]. CVD shows a low water solubility of 0.00444 mg/mL with an average bioavailability of 25% due to a high first-pass effect estimated at 60–75% when administered orally. Interestingly, the resulting metabolites show high antioxidant activity [9].

Due to the long path and high cost of discovering and developing new therapeutic molecules, the trend is observed to improve the physicochemical properties of API with a known profile of action [10,11]. In drugs belonging to the 2 BCS group, the research aims to improve solubility, thus increasing bioavailability. The most commonly used methods of improving solubility include creating formulations with excipients such as biopolymers and reducing API molecules’ size [12,13]. However, the phenomenon of amorphization is being used more and more often [14]. Changing the crystalline structure into an energetically unstable amorphous form often allows for an improvement of solubility. The amorphization process entails technical problems such as API instability or fast recrystallization. Despite significantly better physicochemical properties, the following problems limit using the amorphous form in commercial use [15]. Amorphization as a process is usually considered a good path to improve solubility. However, the literature on the subject indicates that changing the crystalline form to an amorphous API leads to worse physicochemical properties. Adverse changes have been observed for substances such as ritonavir and valsartan [16,17,18]. The resulting disadvantages are attributed to forming a hard film on the API surface, reducing solvent penetration, thereby drastically reducing solubility. An essential aspect of the development of formulations containing amorphous forms is the proper selection of excipients, ensuring the stability of the amorphous form by creating a spatial hindrance limiting the mobility of API molecules [19,20,21]. Selected polymers can also provide a protective form, limiting the access of water molecules starting the recrystallization process [22,23,24]. In addition, many polymers can modify by improving or delaying the release rate of API from the matrix [25,26]. Due to the proper selection of excipients, the stability of the amorphous form, extending its usefulness, can be ensured, and the API availability can be improved [27,28]. An example of such a substance is Soluplus^®^, which provides the stability of the amorphous form while improving the dissolution rate [29].

As part of this work, the research aimed to develop a simple method of obtaining the amorphous form of CVD with the simultaneous development of solid dispersions of the obtained form with biopolymers to stabilize and improve the physicochemical properties. Obtaining amorphousness was confirmed by powder X-ray diffraction (PXRD), differential scanning calorimetry (DSC) techniques as well as Fourier-transform infrared spectroscopy (FT-IR). The obtained amorphous form was stabilized with the selected biopolymers in the kneading process. Changes in dissolution rate and permeability of amorphous form were assessed as effects transition of CVD crystalline form to amorphous form.

## 2. Results and Discussion

The first part of the study involved obtaining the amorphous carvedilol phosphate by grinding CVD in a ball mill. After each 15 min grinding step, a sample was taken to determine the minimum number of cycles necessary to obtain an amorphous form. The interruptions in the milling process were to reduce the temperature generated in the process to prevent the melting of the API. The conducted research allowed us to determine that four 15-min cycles are sufficient to obtain the amorphous form of CVD. The process was continued in the subsequent four cycles to determine the crystalline transition’s improvement to amorphous form; no difference was observed that would justify the use of more than four cycles in the amorphization process. The identity of the obtained samples of CVD after the grinding process was confirmed by powder X-ray diffraction (PXRD), differential scanning calorimetry (DSC), and supported by FT-IR spectroscopy coupled with DFT calculations. PRXD was used to confirm the crystalline form in the starting material.

**Figure 1:**
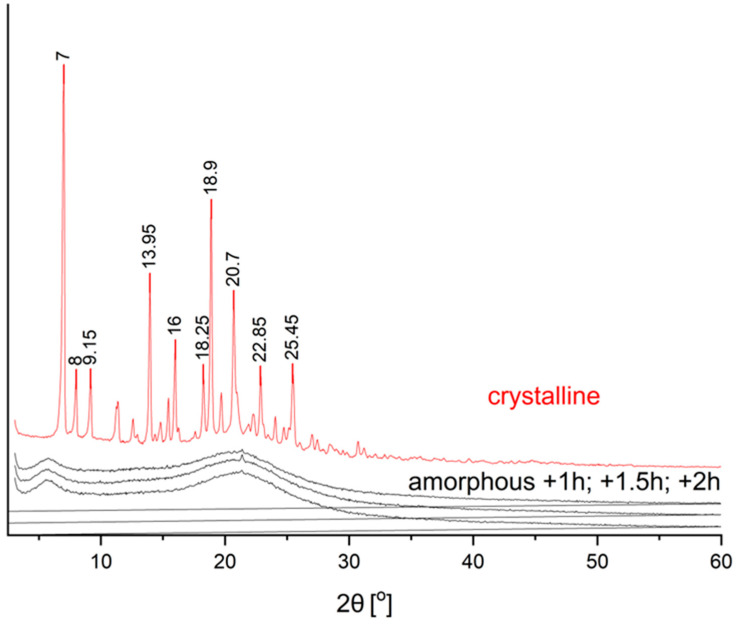
Powder X-ray diffractograms of the solid samples of CVD before and after the ball milling process (1 h, 1.5 h, and 2 h).

The study confirmed the crystalline structure of the CVD in the starting material, thanks to observing characteristic diffraction peaks at: 7.0°; 8.0°; 9.15°; 13.95°; 16.0°; 18.25°; 18.9°; 20.7°; 22.85°; and 25.45° 2θ [30]. Based on the obtained diffractograms, we can observe the disappearance of Bragg reflections and obtaining a characteristic “halo” spectrum, proving the amorphous structure of API [31]. (Figure 1). The process’s length did not affect the quality of the conducted amorphization process. All samples for which process were carried out for at least one hour, samples with an amorphous structure were obtained, confirmation was obtained due to the maintenance of the “halo” effect and the disappearance of the characteristic peaks for the amorphous form of CVD. The obtained results allow stating that the applied method is suitable for the full transformation of CVD to the amorphous form without crystalline grains, which may adversely affect the stability of the obtained amorphous form [32,33]. Optimization towards the CVD amorphization process shortening allows for potential economic use on a larger scale, ensuring optimal process efficiency. However, the PXRD examination alone is not sufficient to confirm the identity of the amorphous form due to the possibility of the formation of a microcrystalline structure [34]. Therefore, a DSC test was performed to confirm the obtained results.

**Figure 2:**
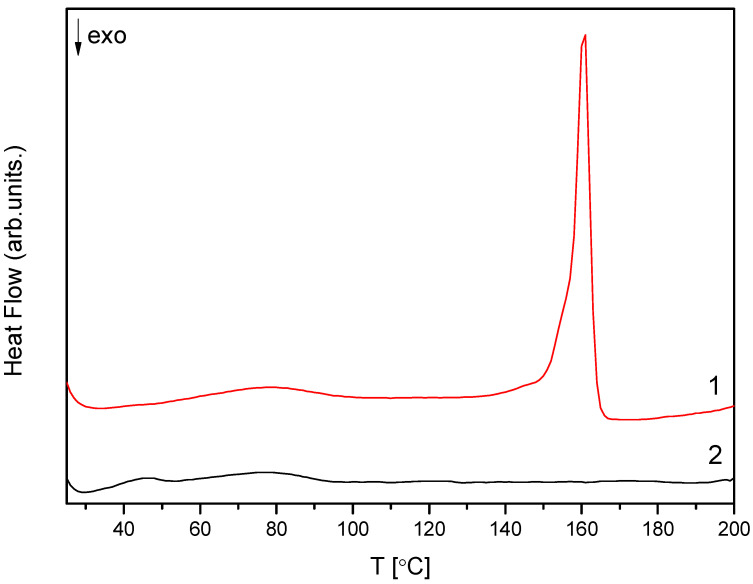
DSC thermograms of the CVD in crystalline (1) and amorphous form (2).

The obtained amorphous material was subjected to DSC testing to confirm the original crystal form’s structural change. The obtained results determined the melting point for CVD in the crystalline form at 160.5 °C (Figure 2). According to previous literature reports, the Thermogram of the amorphous form did not show the melting transition and showed a broad endothermic peak at 46.4 °C, corresponding to its glass transition temperature (Figure 2) [35,36,37,38]. This result confirms the complete amorphization process for the selected methodology and the relatively low humidity of the samples [39], which is extremely important for the amorphous form due to the induction of the recrystallization process by water molecules [40].

The obtained results show that the amorphous form of carvedilol phosphate was obtained only in the grinding process itself, without adding a stabilizing polymer. However, the possibility of high drug load carvedilol even in a 1:1 ratio is possible, proving the relative ease of transition from crystalline to amorphous [29]. However, it should be remembered that polymer addition will always cause additional spatial hindrance, slowing down or stopping the recrystallization process [41,42,43]. The addition of a polymer matrix may also limit the access of water molecules, which is a prerequisite for initiating the recrystallization process [44].

**Figure 3:**
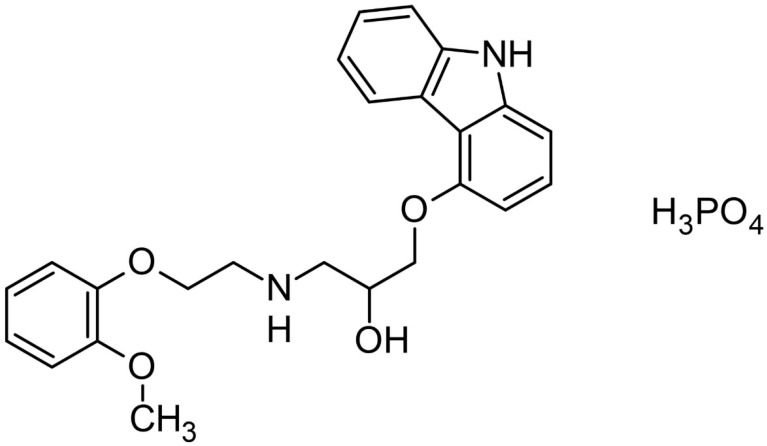
The structural formula of carvedilol phosphate.

The structural formula of CVD is shown in Figure 3. In the FT-IR spectra of the crystalline form of carvedilol phosphate we can assign bands to a given chemical group, and so for 3-[2-(2-methoxyphenoxy) ethylamino]-propan-2-ol structure, most characteristic are the bands associated with the O-H bending (520 cm^−1^), C-O stretching + ”breathing ring” + C-H rocking and wagging in the whole molecule (1024 cm^−1^), C-C stretching+C-H rocking and wagging in the whole molecule (1098 cm^−1^), C-C stretching+C-H wagging in the whole molecule (1126 cm^−1^), C-N stretching (1180 cm^−1^), C-O-H bending (1222 cm^−1^), C-H twisting (1254 cm^−1^), C-C stretching (1331 cm^−1^, 1505 cm^−1^). In the range of 2800–3500 cm^−1^ are visible the bands associated with the C-H stretching (2843 cm^−1^, 2922 cm^−1^, 3063 cm^−1^, 3175 cm^−1^ and 3427 cm^−1^), O-H stretching and wagging+ N-H stretching (3427 cm^−1^). Besides this, there are less intense bands at 728 cm^−1^ (C-H bending out of plane), 798 cm^−1^ (C-H rocking), 849 cm^−1^ (N-H bending out of plane +C-H rocking), 902 cm^−1^ (C-H bending out of plane+N-H bending out of plane), 1047 cm^−1^ (C-O stretching+C-H rocking and wagging in the whole molecule), 1285 cm^−1^ (C-O-H bending+C-H rocking), 1305 cm^−1^ (C-H twisting), 1411 cm^−1^ (C-O-H bending), 1586 cm^−1^ (C=C stretching). For 1-(9H-carbazol-4-yloxy) structure most characteristic are the bands associated with the C-C stretching (1024 cm^−1^, 1098 cm^−1^, 1331 cm^−1^, 1505 cm^−1^), C-O stretching (1098 cm^−1^, 1126 cm^−1^, 1505 cm^−1^), C-N stretching (1254 cm^−1^, 1505 cm^−1^), and N-H stretching (3427 cm^−1^). In addition, there are less intense bands at 728 cm^−1^ (C-H bending out of plane), 1305 cm^−1^ (C-C stretching), 1345 cm^−1^ (“breathing ring” + C-N-C bending + C-H rocking), 1411 cm^−1^ (C-N stretching), 1606 cm^−1^ and 1629 cm^−1^ (C=C stretching). For the phosphate group, most characteristics are the bands associated with the O-H wagging (788 cm^−1^), P-O-H bending (973 cm^−1^, 1024 cm^−1^, 1222 cm^−1^), and O-H stretching (3483 cm^−1^). Besides this, there are less intense bands at 751 cm^−1^ (P-O stretching), 798 cm^−1^ (O-H wagging), and 1047 cm^−1^ (P-O-H bending).

FT-IR spectra of crystalline and amorphous carvedilol phosphate were compared with theoretical spectra obtained by applying the density functional theory (DFT) compared with the database. Characteristic bands of CVD are located between 500 and 2000 cm^−1^ wavelength. The GaussView program was used to visualize the obtained spectra.

**Figure 4:**
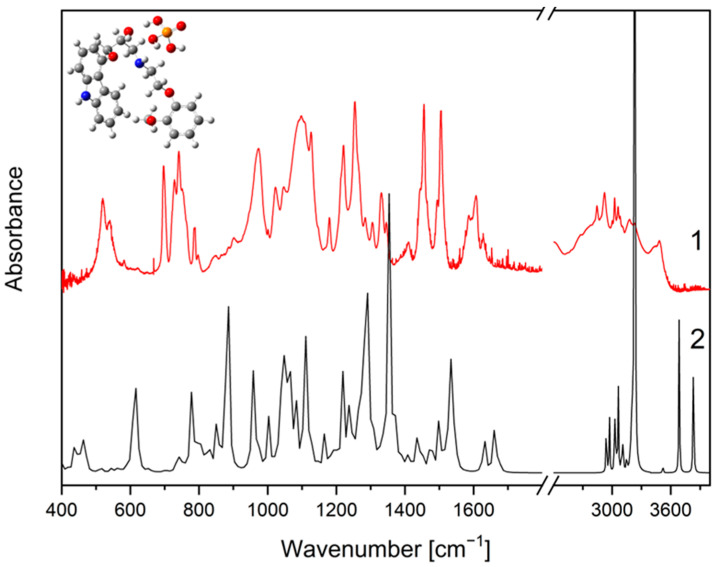
The IR absorption spectra of the CVD: experimental (1) and calculated (DFT) (2) in full range.

**Figure 5:**
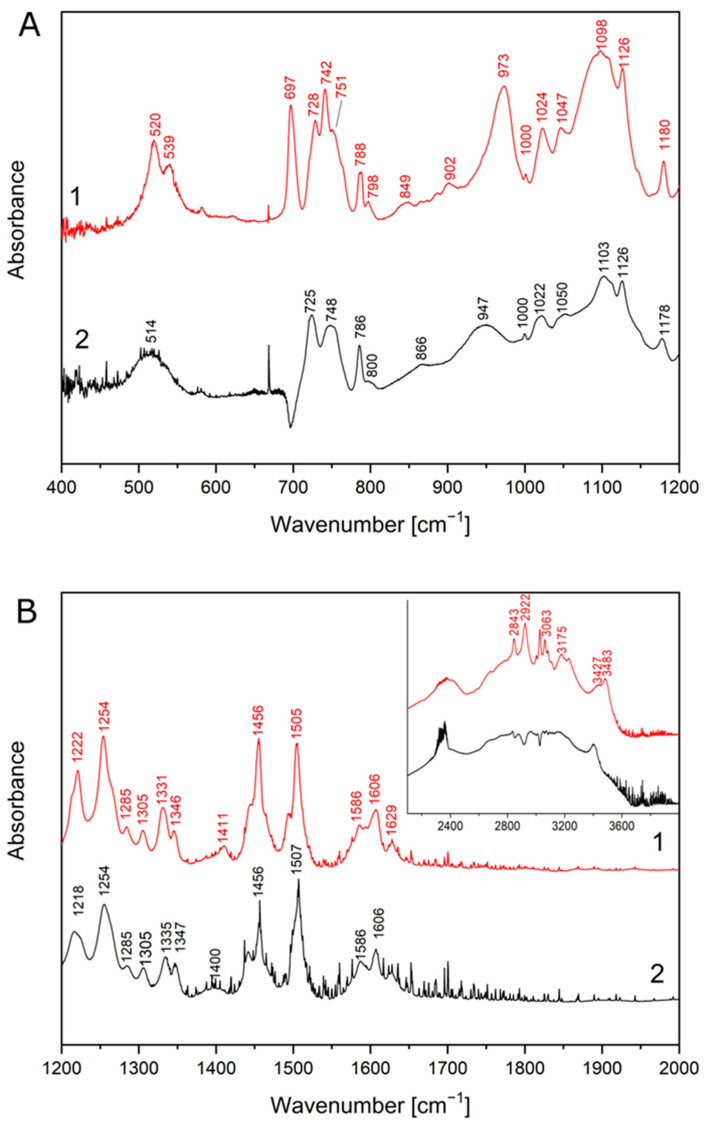
The IR absorption spectra of the CVD: crystalline (1) and amorphous (2) in 400–1200 cm^−1^ (**A**) and 1200–2000 cm^−1^ (**B**).

The obtained FT-IR spectrum of the amorphous form is similar to that published in the patent by Drygas et al., in which the amorphous form of carvedilol phosphate was obtained by spray drying [45]. Comparing the spectrum recorded for the crystalline form with that obtained for the amorphous form (Figure 4), shifts of some bands are visible (crystalline → amorphous, respectively), i.e., 520 cm^−1^ → 514 cm^−1^, 728 cm^−1^ → 725 cm^−1^, 742 cm^−1^ → 748 cm^−1^, 788 cm^−1^ → 786 cm^−1^, 849 cm^−1^ → 866 cm^−1^, 973 cm^−1^ → 947 cm^−1^, 1024 cm^−1^ →1022 cm^−1^, 1047 cm^−1^ → 1050 cm^−1^, 1098 cm^−1^ → 1103 cm^−1^, 1180 cm^−1^ → 1178 cm^−1^ (Figure 5A), 1222 cm^−1^ → 1218 cm^−1^, 1331 cm^−1^ → 1335 cm^−1^, 1346 cm^−1^ → 1347 cm^−1^, 1411 cm^−1^ →1400 cm^−1^, 1505 cm^−1^ →1507 cm^−1^ (Figure 5B). In addition, we observe band disappearance at 539 cm^−1^, 751 cm^−1^ and 902 cm^−1^ due to band widening 520 cm^−1^, 742 cm^−1^, 973 cm^−1^. The amorphous structure spectrum has broadened bands at 514 cm^−1^ and in the range of 800–1000 cm^−1^ and 2500–3500 cm^−1^ (Figure 5A,B). Similar observations were reported by other researchers, pointing out that the amorphous structure may be characterized by the formation of intermolecular systems, a lack of long-range order, and the possibility of the emergence of several molecular conformations [38].

FT-IR spectrum of physical mixture of CVD crystalline and amorphous with all excipients showed no characteristic peaks in any spectra. In addition, there were no new bands observed in CVD-excipients physical mixture, which confirms that no new chemical bonds were formed between the CVD and excipients studied; the obtained spectra of physical mixtures can be found in Supplementary Materials Figures S1–S6.

The condition limiting the bioavailability of drugs belonging to the BCS II class, characterized by good permeability through biological membranes, is their low solubility in the water environment. Carvedilol belonging to this group of drugs shows solubility dependent on the pH of the environment; it dissolves much better in acidic conditions due to the transition to the ionic form, while it precipitates at the reduced pH of the intestine [46]. According to the USP guidelines, the test for carvedilol tablets is performed at acidic pH; however, the conducted tests indicate its highest absorption in the jejunum lumen [47,48,49]. Hence the study was enriched with a release test at pH 6.8. The use of carvedilol phosphate in the formulation of extended-release capsules is also noteworthy; hence, the need to perform the test at a more alkaline pH due to the possibility of obtaining a prolonged release profile due to the polymers used [50].

**Figure 6:**
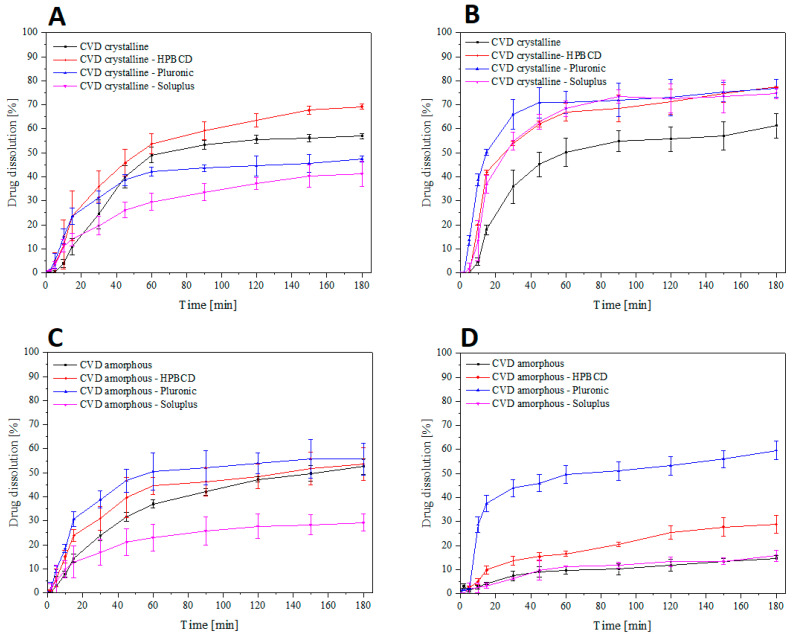
Powder dissolution of CVD from the obtained systems of crystalline CVD at pH 1.2 (**A**), pH 6.8 (**B**), and amorphous CVD at pH 1.2 (**C**) and pH 6.8 (**D**).

The research showed that the amorphous form’s solubility to the crystalline form was worse for the two tested environments at pH 1.2 and 6.8 (Figure 6). The obtained results indicate a worse solubility of CVD in the alkaline environment compared to the acidic environment. In the crystalline form of CVD, we observe significant changes in the release profile after combining polymorphic excipients. In pH 1.2, we observe an improvement in the HPB CD system (Figure 6A). The combination of the CVD crystalline form with the selected polymers showed a significantly higher dissolution rate in the pH environment of ~6.8. (Figure 6B). The obtained amorphous form showed a lower dissolution rate in both pH environments. A significant improvement in the dissolution rate of CVD in the amorphous form was observed for the system containing Pluronic^®^ F-127; for both tested pH conditions, it showed the best improvement in the physicochemical parameters of the system (Figure 6C,D). Despite the significantly improved solubility in the amorphous form of CVD combined with Pluronic^®^ F-127, we still observe worse lower solubility than in the crystalline form. However, the obtained results are close to the crystalline form.

In most cases, we can expect an improvement in the solubility of the amorphous form compared to the crystalline form; however, in the literature, we find examples when the obtained amorphous form is characterized by reduced solubility [16,51]. This effect is attributed to forming a hard film on the API surface, limiting solvent wetting and thus reducing the API contact surface. The observed improvement in solubility in combination with Pluronic^®^ F-127 can be attributed to the dispersion of amorphous API, eliminating the physical hindrance of the hard, solvent-impermeable API surface. Improved wettability translates into more significant CVD contact with the medium used, significantly affecting the obtained dissolution rate [52].

The polymers used to improve solubility in the obtained physical mixtures are an essential property to be considered in the conducted study. The literature on the subject indicates no dependence of the release rate on the molar mass of the polymer used with carvedilol phosphate [29,53]. However, higher solubility is expected for more soluble polymers in a given medium, releasing API from the formulation. Hydroxypropyl-β-cyclodextrin, for example, shows a clear relationship between the increase in release rate and pH with the best improvement for pH 4; however, an improvement in solubility is observed over the entire pH range [29]. It was shown that Pluronic^®^ F-127 showed a delay in release at acidic pH; however, at alkaline pH, it showed a significant improvement in API solubility, which was confirmed in the tests performed [54]. On the other hand, Soluplus shows the lowest solubility in acidic pH and the highest in alkaline pH; in the study, it was shown that the systems containing the polymer showed better solubility at higher pH, which confirms the assumption that the solubility of the entire system will depend on the solubility of the polymer used to compose the physical mixture [55,56]. The obtained results indicate the potential development of formulations containing carvedilol as an active substance based on selected polymers, allowing API release in a selected pH-dependent environment.

**Figure 7:**
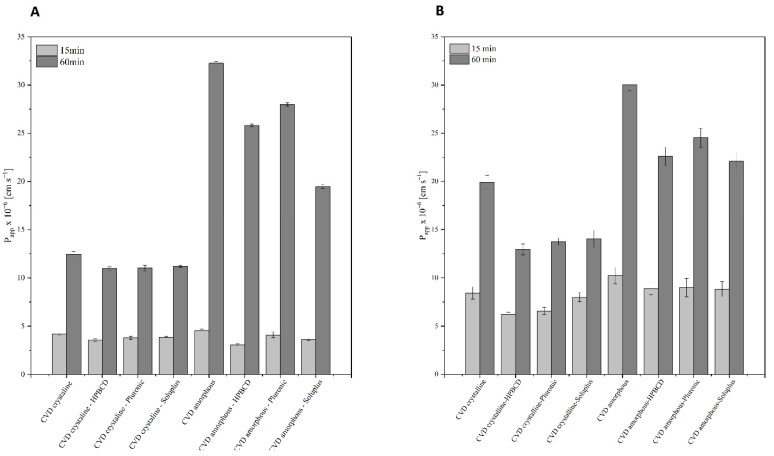
Permeability of crystalline and amorphous CVD and their solid dispersion at pH 1.2 (**A**) and pH 6.8 (**B**).

Carvedilol phosphate belongs to the 2nd BCS group characterized by poor solubility, limiting bioavailability, and good permeability through the biological membrane system. Calculated apparent permeability coefficients of CVD in crystalline and amorphous forms with excipients are collected in Figure 7. The permeability study was carried out in the PAMPA-GIT model for pH 1.2 and 6.8 correspondings to subsequent gastrointestinal tract sections; the study was conducted for 3 h. The permeation study indicates a much higher permeability of CVD in amorphous form for both environments [57]. Moreover, the improved permeability obtained was maintained with the addition of excipients. The obtained results indicate higher permeability of CVD in the environment with a higher pH for all the presented systems, regardless if API is in the crystalline or amorphous form [58]. Although the addition of excipients causes a slight reduction in permeability compared to the pure form of CVD used in the study, the model used may impact the obtained result. PAMPA is only a simplified model of in vitro permeability, taking into account only passive diffusion, which, however, is significantly reflected in vivo conditions [59]. Despite the improved permeability for the resulting amorphous form, both forms of CVD are characterized as highly permeable (*Papp* > 1 × 10^−6^ cm/s) [60].

We observe a significant dependence deviating from the classical approach in the conducted study, the interplay between solubility and permeability [61]. The best-soluble systems do not show the best permeability in the PAMPA-GIT model. The obtained results confirm the latest approach to the mutual dependence of solubility and permeability, which indicates that it is not a linear relationship, and conclusions about the improvement of bioavailability should not be drawn solely based on partial results [61]. The literature on the subject indicates frequent examples of reduced permeability for systems with improved solubility [62,63,64]. This mechanism has not been fully explored [65]. However, it is attributed to the interaction of API molecules with the cell membrane and the polymers used and their interaction with API molecules and layer of immiscible medium on the surface of the biological membrane [66]. Moreover, it should be taken into account the simplification of the applied model based only on passive diffusion; the reduction of permeability may result from clogging of the membrane pores by the polymers used [67,68], conditioning the effect of lowering permeability for the solid CVD dispersions of excipients to the pure API in a crystalline or amorphous form.

Another potential cause of improved permeability despite decreasing solubility is obtaining a supersaturated solution. The conducted studies allow for the conclusion that the permeability of API increases with the saturation of the solution, thus testing different forms of the same API at the same concentration but with different solubility will generate a result proving higher permeability of less soluble API due to higher saturation of the solution or supersaturation. Due to the concentration close to or exceeding the solubility, API penetrates more efficiently through the biological membrane system in passive diffusion to achieve an energetically beneficial equilibrium state [69,70,71].

## 3. Materials and Methods

### 3.1. Materials

Carvedilol phosphate (99% purity was supplied by Polpharma Starogard Gdański (Starogard Gdański, Poland), while excipients: β-cyclodextrin, Pluronic^®^ F-127 (Poloxamer 407) were obtained from Sigma Aldrich Chemie (Berlin, Germany) and Soluplus^®^ (PVCL-PVA-PEG) from BASF ChemTrade GmbH (Ludwigshafen, Germany). Acetonitrile of super purity was supplied by Romil (Cambridge, England) and formic acid (98–100%) by POCH Gliwice (Gliwice, Poland). The USF T-801 water purification system (Vienna, Austria) and Exil SA 67120 millipore purification system (Burlington, USA) prepared high-quality pure water. Hydrochloric acid, sodium bromide, and sodium chloride were obtained from POCH Gliwice (Gliwice, Poland).

### 3.2. Preparation of the Amorphous Form of Carvedilol Phosphate

The starting material for the tests was the crystalline form of carvedilol phosphate. The crystalline structure of CVD to an amorphous one was carried out by grinding in a ball mill Retsch MM 400 (Retsch GmbH, Haan, Germany). A sample was ground in 8 cycles of 15 min with 5-min breaks to prevent CVD salt melting by the temperature generated in the process. During grinding, the mill’s operating frequency was 30 Hz. Based on identity research, it was determined that 4 milling cycles lasting 15 min are enough to obtain an amorphous form. The required amount of amorphous CVD was prepared for each test to prevent the recrystallization process.

### 3.3. Powder X-ray Diffraction (PXRD)

PXRD analysis was performed at ambient temperature using the Bruker D2 Phaser (Bruker, Billerica, MA, USA) diffractometer with LynxEye XE-T 1-dim detector and Cu Kα radiation (λ = 1.54056 Å, generator setting: 40 kV and 40 mA). Diffraction data were collected at the 2θ scanning range between 5° to 40° with a step size of 0.02° and a counting time of 2 s/step.

### 3.4. Different Scanning Calorimetry (DSC)

DSC analysis of all compounds was performed using a DSC 8500 Perkin Elmer equipped with an intercooler system (PerkinElmer, Waltham, MA, USA). Indium was used for calibration. Accurately weighed samples were placed in 60 μL sealed cells and heated at a scanning rate of 10 °C min−1 from 0 °C to 200 °C under a nitrogen purge gas with a flow rate of 20 mL min−1. Each run was repeated at least twice.

### 3.5. FT-IR Spectroscopy and Density Functional Theory (DFT)

Pellets of crystalline and amorphous forms of CVD were prepared with IR grade KBr (1 mg of API: 300 mg of KBr). Reference samples were prepared for API complexes with excipients in a hydraulic press, exerting 8 tons by 15 min. Spectrum was obtained at the range 400 and 4000 cm^−1^, using an FT-IR Bruker IFS 66v/S. Accurate spectra analysis was made possible by identifying band types, their intensity, and location to the appropriate theoretical spectra obtained due to quantum-chemical calculations using electron density theory (DFT) with a computational basis. The GaussView program version 6.0 was used to visualize the received spectra.

The DFT calculations with a B3LYP functional and 6–31 G(d,p) as basis set were used as supportive methods.

### 3.6. Preparation of Physical Mixtures of CVD with Excipients

Solid dispersions of carvedilol with hydroxypropyl-β-cyclodextrin (HP-β-CD), Pluronic^®^ F-127, and Soluplus^®^ were obtained for the crystalline and amorphous forms by grinding API with the polymer in an agate mortar; the process was carried out for 30 min. The weight ratio of CVD to biopolymer was 1:1.

### 3.7. UHPLC-DAD Method

Changes in CVD concentration during the dissolution rate and permeability tests were determined using the developed UHPLC-DAD method. Chromatographic separation was possible by using a liquid chromatography system (Dionex Thermoline Fisher Scientific, Dreieich, Germany) equipped with a high-pressure pump (UltiMate 3000), an autosampler (UltiMate 3000), and a DAD detector (UltiMate 3000) with Chromeleon software version 7.0 from Dionex Thermoline Fisher Scientific (Dreieich, Germany). The method was based on Kinetex^®^ C18 100 Å, (Torrance, CA, USA) (100 × 2.1 mm, 2.6 µm) using a mobile phase composed of 0.1% formic acid-acetonitrile (50:50 *v*/*v*) at a flow rate of 0.3 mL/min. The injection volume was 5.0 µL, peaks were observed at 30 °C after 1 min using the wavelength of detection set at 240 nm. The chromatographic method was validated by simultaneous analysis of samples according to the International Conference on Harmonization (ICH) Q2(R2). Before analysis, the samples were filtered with a 0.2 µm membrane syringe filter.

### 3.8. Apparent Solubility Studies

The apparent solubility studies of amorphous and crystalline CVD forms and their solid dispersions with excipients were carried out using an Agilent 708-DS Dissolution Apparatus (Agilent Technologies, Santa Clara, CA, USA). Conditions of the dissolution method were temperature 37 °C ± 0.5 and 50 RPM.

Obtained systems (weight ratio carvedilol: excipient 1:1) were weighed into gelatin capsules. The capsules were placed in sinkers to prevent the floating of the capsule before its disintegration. The capsules were placed in 500 m of media that simulated gastric acid at pH 1.2 (0.1 mol/dm^3^ HCL) and intestine at pH 6.8 phosphate buffer. The samples (volume: 5 mL) were collected at specific time points throughout the entire study, simultaneously replacing the equal volume of specific, temperature-equilibrated media. Samples after collection were filtered through a 0.2 μm membrane syringe filter.

The similarity of dissolution profiles of amorphous and crystalline forms of carvedilol with excipients was established on *f*_1_ and *f*_2_ parameters and was defined by the following equations:

$$f_{1}=\frac{\sum_{j=1}^{n}\left|R_{j}-T_{j}\right|}{\sum_{j=1}^{n}R_{j}}\times 100$$

$$f_{2}=50\times \log\left({\left(1+\left(\frac{1}{n}\right)\overset{n}{\underset{j=1}{\sum }}{\left|R_{j}-T_{j}\right|}^{2}\right)}^{-\frac{1}{2}}\times 100\right)$$

In which *n* is the number of withdrawal points, *R_j_* is the percentage dissolved of reference at the time point *t*, and *T_j_* is the percentage dissolved of the test at the time point t. It is assumed that the release profiles show similarity when the value of *f*_1_ is between 0 and 15, while *f*_2_ assumes a value close to 100 (not less than 50) [72].

### 3.9. Permeability Studies of Systems

The studies of the evaluation of changes in the permeation of substances were carried out using the parallel test of permeability through artificial membranes (PAMPA, Parallel Artificial Membrane Permeability Assay) of gastrointestinal permeation. Received values of apparent permeability were compared using the ANOVA variance analysis. The parallel permeability test through artificial biological membranes (PAMPA) served as an in vitro model of passive intercellular penetration. For testing, permeability solutions were prepared of saturated amorphous and crystalline carvedilol phosphate solutions in systems with excipients in a 1:1 weight ratio and free form. The prepared solutions were filtered through 0.2 μm pore size filters.

The PAMPA test consists of 96-well filter microplates, divided into donor and acceptor chambers, separated by a 120 μm microfilter, and coated with a 20% lecithin solution. The plates were incubated at 37 °C for 60 min minutes. After incubation in the donor and acceptor part, the carvedilol phosphate concentration was determined using the UHPLC-DAD method. The assessment of the effect of a substance on penetration was determined by comparing the values apparent permeability coefficients using equations:

$$P_{app}=\frac{-\ln\left(1-\frac{C_{A}}{C_{equilibrium}}\right)}{S\times \left(\frac{1}{V_{D}}+\frac{1}{V_{A}}\right)\times t}$$

where *V_D_*—donor volume, *V_A_*—acceptor volume, $C_{equilibrium}=\frac{C_{D}\times V_{D}+C_{A}\times V_{A}}{V_{D}+V_{A}}$, *S*—membrane area, *t*—incubation time (in seconds).

Compounds with a *P_app_* < 1 × 10^−6^ cm/s are classified as low permeability, and compounds with a *P_app_* > 1 × 10^−6^ cm/s are classified as ones with high permeability [73].

## 4. Conclusions

PXRD, DSC, and FT-IR spectroscopy confirmed the identity of the obtained amorphous form and the original crystalline structure of CVD. No differences were observed between the grinding time with at least four cycles and the quality of the obtained amorphous form. The obtained solid dispersions with hydrophilic polymers and pure API were subjected to the dissolution rate test at pH 1.2 and 6.8. The conducted study showed a decrease in the solubility of the obtained amorphous form regarding crystalline form. It has been shown that Pluronic^®^ F-127 in combination with CVD can form the basis for further development of new formulations due to a significant improvement in the dissolution rate of the resulting amorphous form, bringing it closer to the more soluble crystalline form for both tested pH environments. The permeability study was carried out in the PAMPA-GIT model for pHs corresponding to the dissolution rate study in the last stage. The obtained results indicate a significantly improved permeability for the amorphous form, despite its lower solubility, and a positive relationship between the duration of the test and the permeability of API was also shown.

The conducted research shows the ratio of a rare phenomenon of lowering the solubility of the amorphous form of CVD and is the basis for further research to determine the mechanisms behind this process. The obtained results allow for the potential use of the obtained solid dispersions in further formulation research.

## Data Availability

Data is contained within the article and Supplementary Materials.

## Supplementary Materials

The following are available online, Figure S1: FT-IR spectrum of crystalline CVD—HPCD physical mixture; Figure S2: FT-IR spectrum of crystalline CVD—Soluplus^®^ physical mixture; Figure S3: FT-IR spectrum of crystalline CVD—Pluronic^®^ F127 physical mixture; Figure S4: FT-IR spectrum of amorphous CVD—HPCD physical mixture; Figure S5: FT-IR spectrum of amorphous CVD—Soluplus^®^ physical mixture; Figure S6: FT-IR spectrum of amorphous CVD—Pluronic^®^ F127 physical mixture; Figure S7: Amorphous CVD diffractogram—HpB CD physical mixture; Figure S8: Amorphous CVD diffractogram—Soluplus^®^ physical mixture; Figiure S9: Amorphous CVD diffractogram—Pluronic^®^ F127 physical mixture.

## Sample Availability

Samples of the compounds are not available from the authors.
