# Supplementary material for: Amorphous Form of Carvedilol Phosphate—The Case of Divergent Properties
Source: Molecules. 2021 Sep 1;26(17):5318. doi: 10.3390/molecules26175318 (PMC8434513; doi:10.3390/molecules26175318)
Supplement: Supplementary file 1 [file molecules-26-05318-s001.zip › molecules-1311268-supplementary.pdf]

molecules MDPI

Supplementary materials

Amorphous form of carvedilol phosphate - The case of divergent properties

Szymon Sip [1], Natalia Rosiak [1], Andrzej Miklaszewski [2], Patrycja Talarska [3], Ewa Dudziec [4] and Judyta Cielecka-Piontek [1,*]

[1] Department of Pharmacognosy, Poznan University of Medical Sciences, 4 Swiecickiego Street, 60-781 Poznan, Poland; szymonsip@ump.edu.pl (S.S.),nrosiak@ump.edu.pl (N.R.), jpiontek@ump.edu.pl (J.C.P.)
[2] Institute of Materials Science and Engineering, Poznan University of Technology, Jana Pawła II 24, 61-138 Poznan, Poland, andrzej.miklaszewski@put.poznan.pl (A.M.)
[3] Department of Immunobiology, Poznan University of Medical Sciences, ul. Rokietnicka 8, 60-806 Poznań, Poland; patrycjatalarska@ump.edu.pl (P.T.)
[4] Department of Rheumatology and Rehabilitation, Poznan University of Medical Sciences, ul. 28 Czerwca 1956 r. 135/147, 61-545 Poznań, Poland; ewa.dudziec@ump.edu.pl (E.D.)
* Correspondence: jpiontek@ump.edu.pl

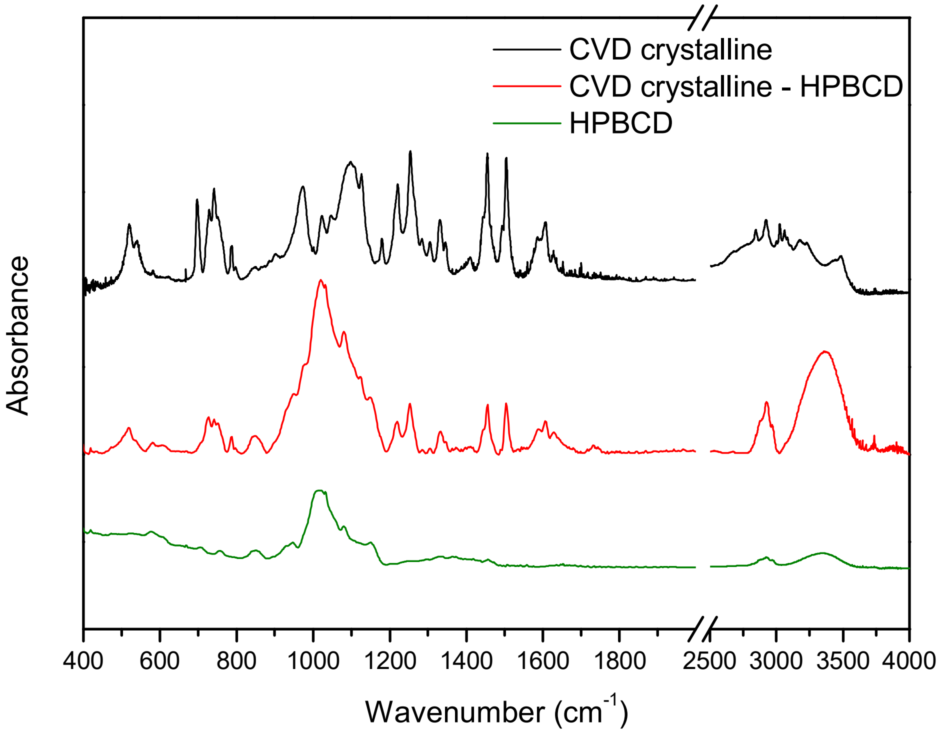

Figure S1. FT-IR spectrum of crystalline CVD - HPBCD physical mixture.

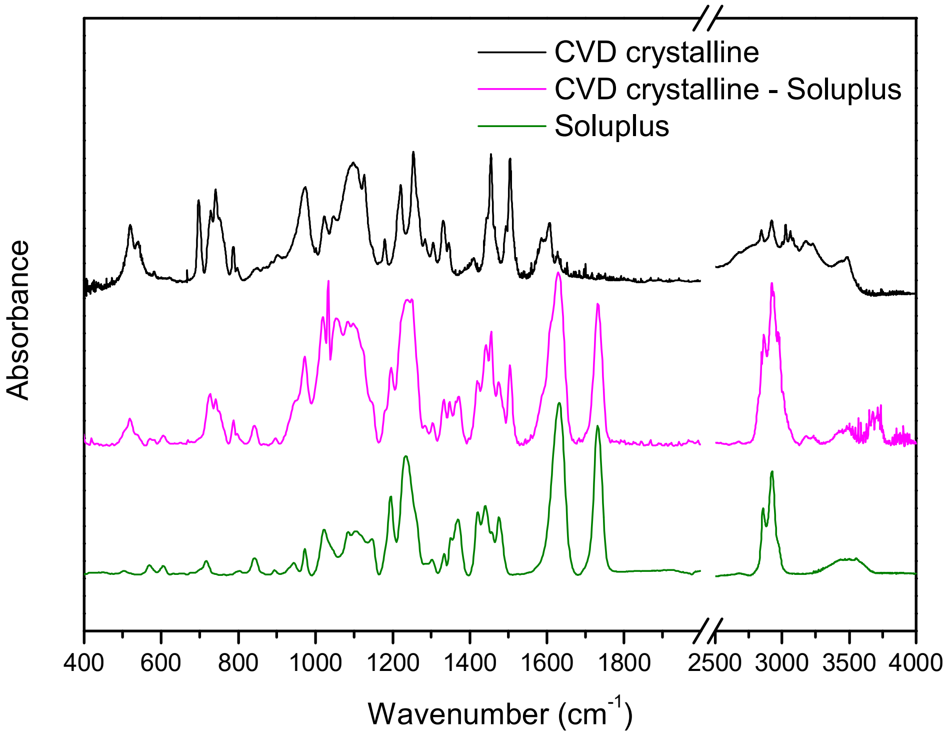

Figure S2. FT-IR spectrum of crystalline CVD – Soluplus® physical mixture.

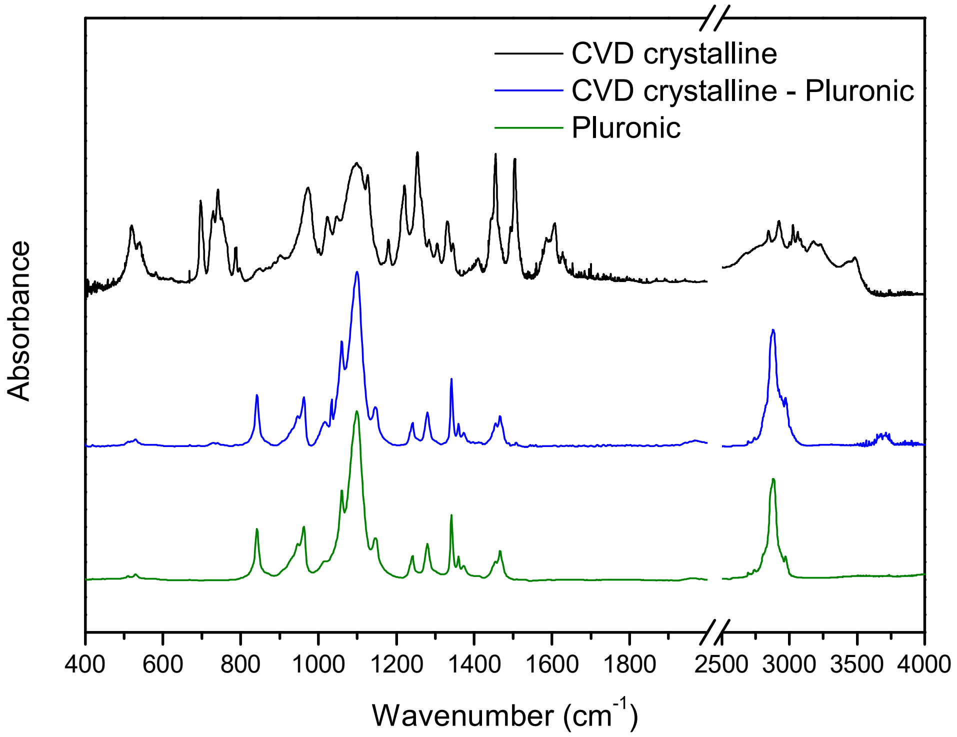

Figure S3. FT-IR spectrum of crystalline CVD – Pluronic® F127 physical mixture.

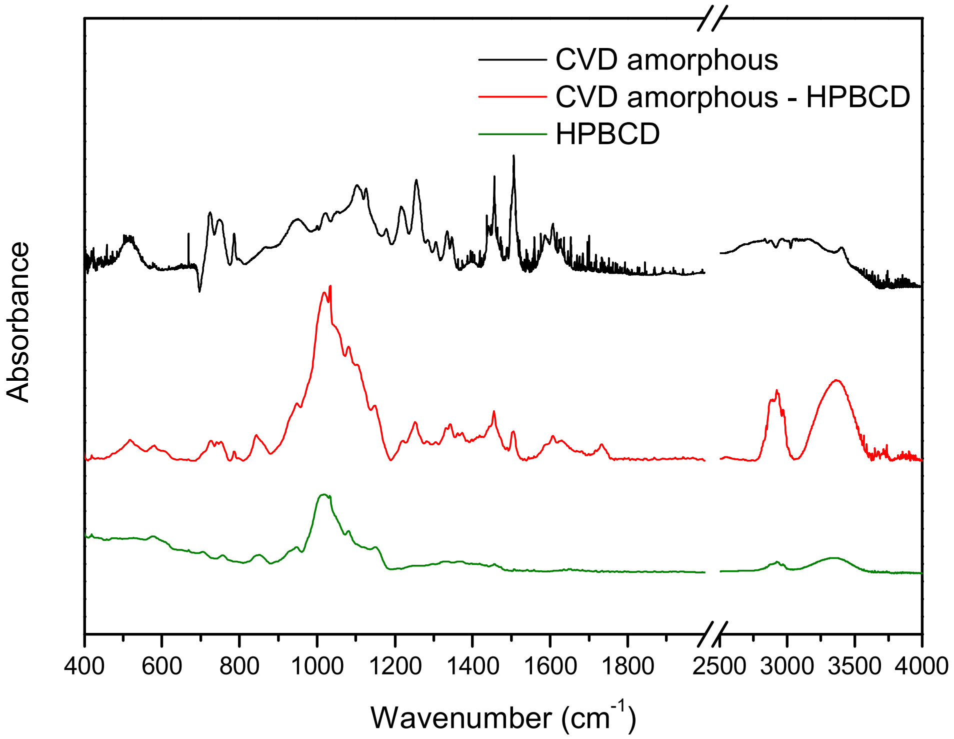

Figure S4. FT-IR spectrum of amorphous CVD - HPBCD physical mixture.

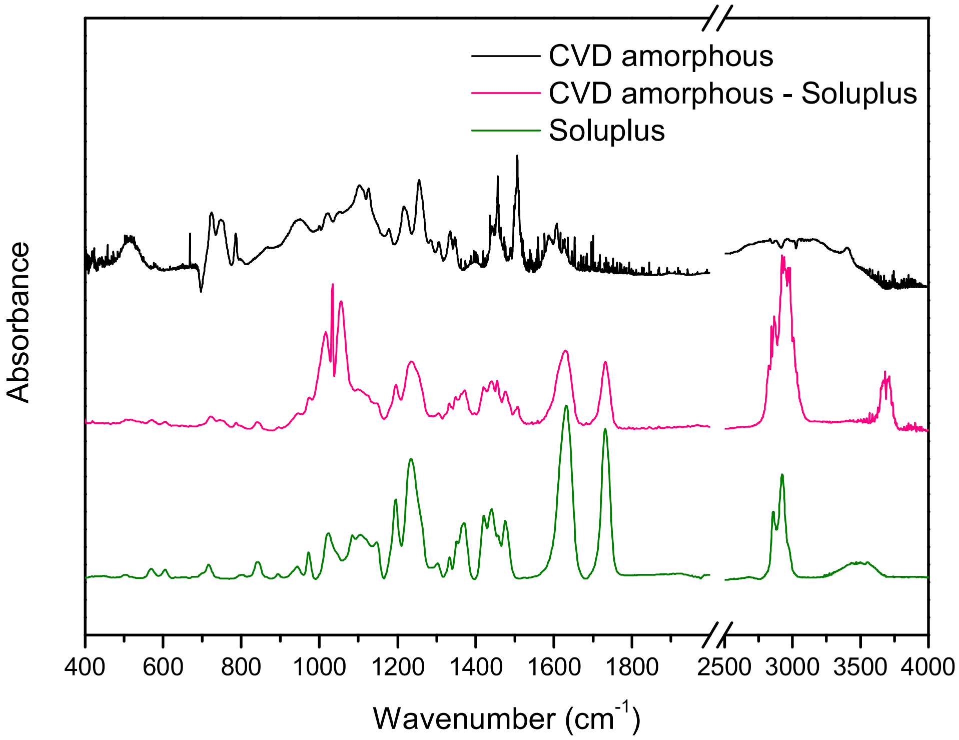

Figure S5 FT-IR spectrum of amorphous CVD – Soluplus® physical mixture.

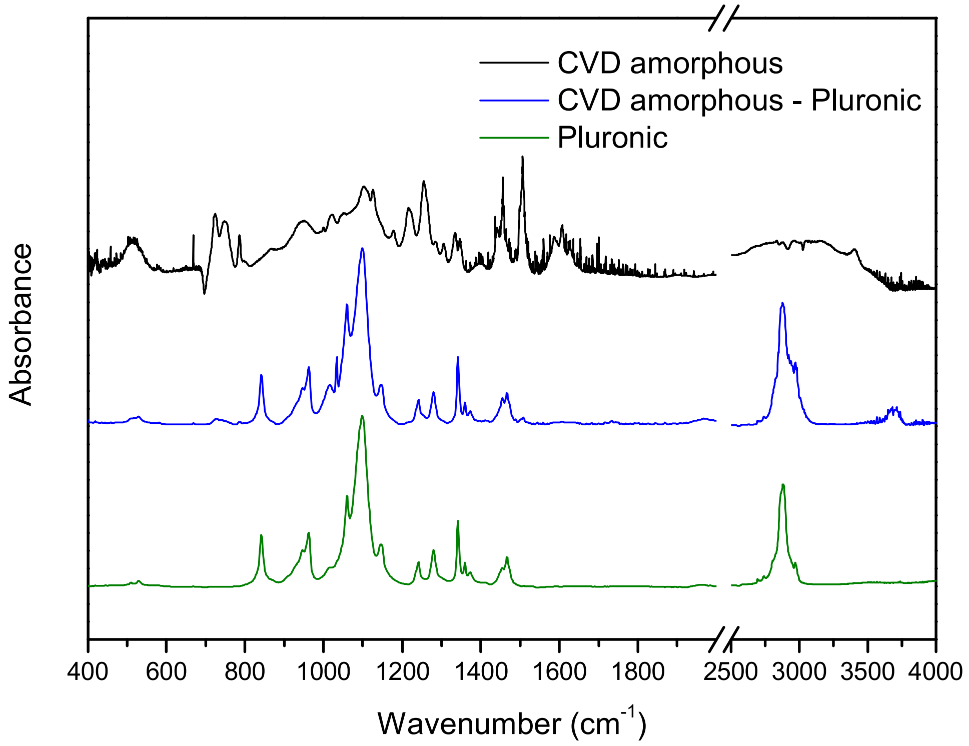

Figure S6. FT-IR spectrum of amorphous CVD – Pluronic® F127 physical mixture.

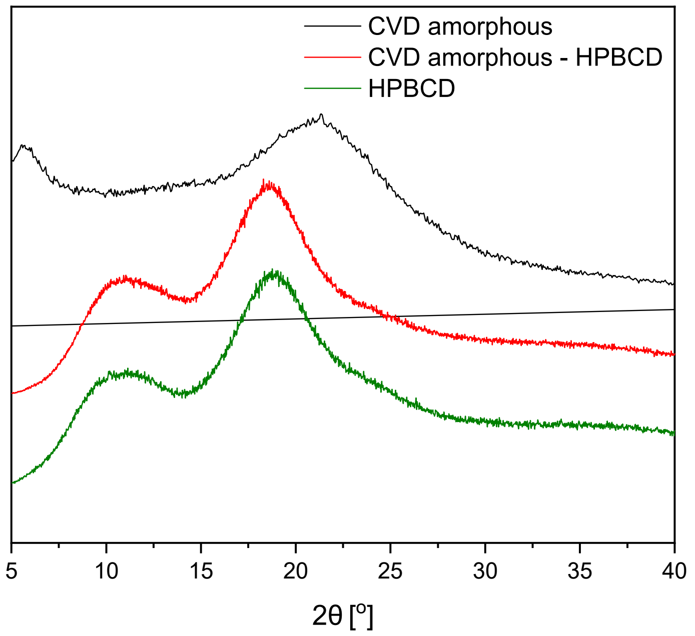

Figure S7. Amorphous CVD diffractogram – HPBCD physical mixture.

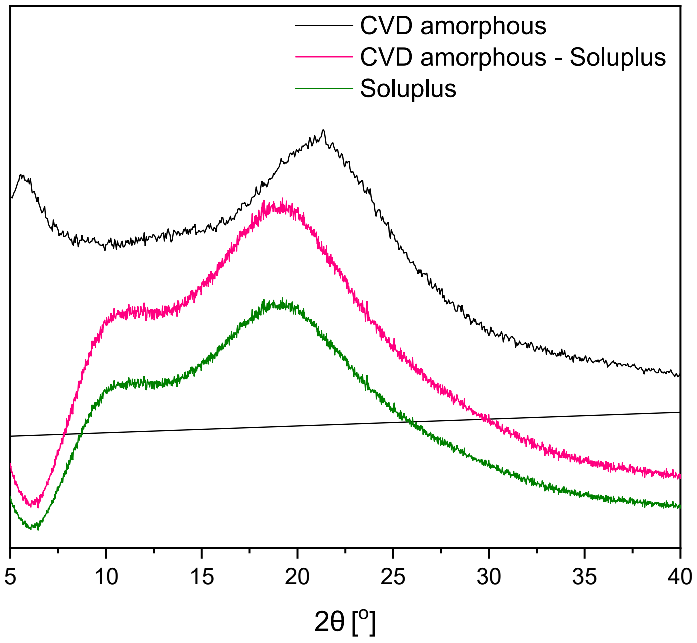

Figure S8. Amorphous CVD diffractogram – Soluplus® physical mixture.

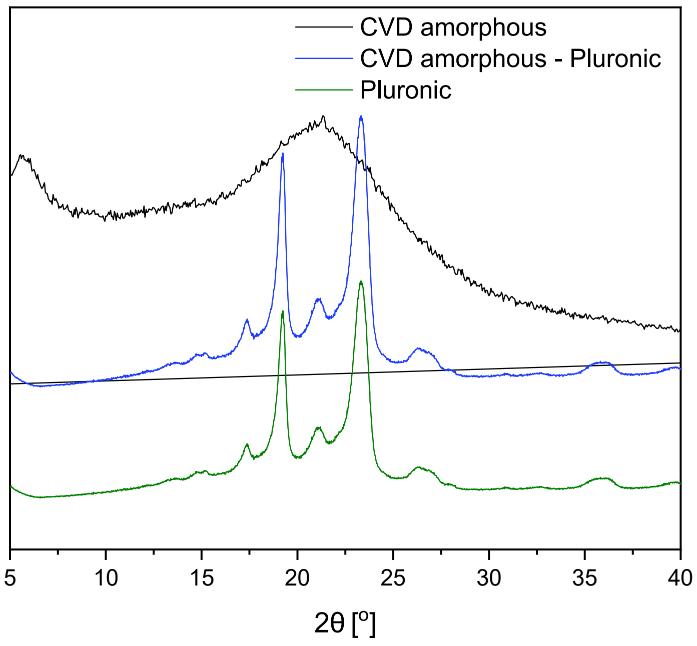

Figure S9. Amorphous CVD diffractogram – Pluronic® F127 physical mixture.
